# PGE2-JNK signaling axis non-canonically promotes Gli activation by protecting Gli2 from ubiquitin-proteasomal degradation

**DOI:** 10.1038/s41419-021-03995-z

**Published:** 2021-07-15

**Authors:** Jun Yang, Juan Wang, Yuan Liu, Yu Zhang, Wenjing Huang, Yu Zou, Yanyan Qiu, Weiyang Cai, Jing Gao, Hu Zhou, Yingli Wu, Weijun Liu, Qingqing Ding, Yanjie Zhang, Pei-hao Yin, Wenfu Tan

**Affiliations:** 1 Department of Pharmacology, School of Pharmacy, Fudan University, 201203 Shanghai, China; 2 Department of General Surgery, Putuo Hospital, Shanghai University of Traditional Chinese Medicine, 200062 Shanghai, China; 3 Department of General Surgery, Shanghai Putuo Central School of Clinical Medicine, Anhui Medical University, 230601 Hefei, Anhui China; 4 Department of Oncology, Shanghai 9th pepople’s Hospital, Shanghai Jiao Tong University School of Medicine, 280 Mohe Road, 201999 Shanghai, China; 5 Department of Analytical Chemistry and CAS Key Laboratory of Receptor Research, Shanghai Institute of Materia Medica, Chinese Academy of Sciences, 201203 Shanghai, China; 6 Hongqiao International Institute of Medicine, Shanghai Tongren Hospital/Faculty of Basic Medicine, Chemical Biology Division of Shanghai Universities E-Institutes, Key Laboratory of Cell Differentiation and Apoptosis of the Chinese Ministry of Education, Shanghai Jiao Tong University School of Medicine, Shanghai, China; 7 Department of Radiation Oncology, Washington University School of Medicine, St Louis, MO 63108 USA; 8 Department of pathology, University of Texas MD Anderson Cancer Center, Houston, TX 77030 USA

**Keywords:** Cell signalling, Molecular biology

## Abstract

Both bench and bedside investigations have challenged the supportive role of Hedgehog (Hh) activity in the progression of colorectal cancers, thus raising a critical need to further deeply determine the contribution of Hh to the growth of colorectal cancer. Combining multiple complementary means, including in vitro and in vivo inflammatory colorectal cancer models, and pathological analysis of clinical colorectal cancer patients samples. We report that colorectal cancer cells hijack prostaglandin E2 (PGE2) to non-canonically promote Hh transcriptional factor Gli activity and Gli-dependent proliferation of colorectal cancer cells in a Smo-independent manner. Mechanistically, PGE2 activates c-Jun N-terminal kinase (JNK), which in turn enables Gli2 to evade ubiquitin-proteasomal degradation by phosphorylating Gli2 at Thr1546. This study not only presents evidence for understanding the contribution of Hh to colorectal cancers, but also provides a novel molecular portrait underlying how PGE2-activated JNK fine-tunes the evasion of Gli2 from ubiquitin-proteasomal degradation. Therefore, it proposes a rationale for the future evaluation of chemopreventive and selective therapeutic strategies for colorectal cancers by targeting PGE2-JNK-Gli signaling route.

## Introduction

The Hedgehog (Hh) signaling pathway, which is evolutionarily conserved across organisms ranging from insects to mammals, plays a critical role in embryonic development and tissue regeneration [1]. Binding ligands, including sonic Hh (SHh), Indian Hh (IHh), and desert Hh (DHh), to the twelve transmembrane receptor Ptch relieves the inhibitory effect on Smoothened, another critical receptor of Hh pathway with G protein coupled receptor-like structure. This subsequently causes accumulation of Smo in the primary cilium and ultimately initiates the transcriptional output of zinic finger transcriptional factor Gli, which includes Gli1, Gli2, and Gli3. Among them, Gli2 functions as the primary activator of Hh activity [2, 3]. Postnatal hyperactive Hh signaling activity has been well characterized as a driver contributor to the initiation and progression of various types of cancers, including basal cellular carcinoma, medulloblastoma, and rhabdomyosarcoma. Such cancers harbor aberrant Hh activity commonly caused by genetic alterations in critical components of the pathway, for example, Ptch, Smoothened, Sufu, etc [4–6]. The approval of Hh inhibitors targeting Smo for clinical treatment of advanced basal cell carcinoma further confirms the addiction of this type of cancer to aberrant Hh pathway activity [7].

However, the contribution of Hh pathway activity to their progression remains far from being fully elucidated, such as colorectal cancers which rarely carry genetic changes in Hh genes [5]. It has been initially suggested that Hh pathway activation in an autocrine manner by the upregulated the expression of Hh ligands in colorectal cancer cells promotes the growth and metastasis of colorectal cancers [8, 9]. Many studies have also revealed that stromal Hh pathway activity provoked by Hh ligands secreted by colorectal cancer cells is essential for the carcinogenesis and growth of colorectal cancer, which is reminiscent of the paracrine activation of Hh pathway in mesenchymal cells during intestinal development [10–14]. Unfortunately, these arguments are challenged by the discouraging results from a clinical trial with vismodegib included in first-line therapy of metastatic colorectal cancer [15]. Additional investigations reveal that stromal Hh activity is required for the initiation of intestinal adenoma but not for the subsequent tumor growth, and that the stromal Hh activity is downregulated in colorectal cancer, while its restoration limits cancer growth [16]. Colorectal cancer is a leading cause of cancer related mortality. A large body of evidence strongly indicates that chronic inflammatory stimuli may increase risk of colorectal cancer. Epidemiologic and clinic studies have revealed that non-steroidal anti-inflammatory drugs (NSAIDs) may reduce the risk of colorectal cancer, acting primarily through inhibiting cyclooxygenase-1 (COX-1) and COX-2, two enzymes involved in prostaglandin biosynthesis. COX2 expression has been found to be elevated in colorectal cancers and to be associated with worse survival rate of colorectal cancer patients [17, 18]. Prostaglandin E2 (PGE2) is the most abundant prostaglandins found in colorectal cancers and the major protumorigenic metabolites of inflammatory factor COX2 [18]. Despite multiple molecular mechanisms underpinning how PGE2 promotes the initiation and progression of colorectal cancers have been uncovered [19], the development of selective therapies by targeting PGE2 signaling axis for colorectal cancers, a strategy supposed to be better than targeting COX2 [20, 21], makes no significant progress so far. This highlights a critical need to deeper understand the molecular mechanisms behind how PGE2 promotes the initation and progression of colorectal cancer, and to identify novel druggable vulnerability downstream of PGE2 for chemoprevention and selective therapies of colorectal cancers. In this study, we investigated whether transcription factor Gli of Hh pathway may be non-canonically activated by PGE2, thereby promoting the initiation and progression of colorectal cancers.

## Materials and methods

### Cell culture and transfection

All the cell lines used in this study were purchased from the American Type Culture Collection (Manassas, VA), and were routinely cultured in DMEM supplemented with 10% fetal bovine serum (FBS) (Corning Life Sciences) and 1% penicillin/streptomycin/l-glutamine (Corning Life Sciences). Cells were periodically tested to be free of mycoplasma contamination. No cell lines used in this study were found in the database of commonly misidentified cell lines that is maintained by ICLAC and NCBI Biosample.

The plasmids and siRNAs were transfected using Lipofectamine 2000 according to the manufacturer’s instruction.

### Animal studies

All animal studies conformed to our animal protocols approved by the Animal Experimentation Ethics Committee of School of Pharmacy, Fudan University. *APC*^min/+^ mice were purchased from Jackson Laboratory (Bar Harbor, ME). Male *Apc*^min/+^ mice at age of 5 weeks old were treated with vehicle, or PGE2 at 150 μg/100 μl/mouse by gavage twice per day in combination with or without SP600125 at 50 mg/kg by gavage once per day, GDC-0449 at 25 mg/kg by gavage twice per day, JQ1 at 100 mg/kg by gavage once per day for 6 weeks. Each mouse was randomly assigned to each group and the investigator was blinded to the group allocation. After the mice were sacrificed, the polyp number and size were counted and measured under a Nikon SMZ1000 dissecting scope, and were harvested for analyzing the expression of Hh target genes by real time PCR [22].

### Human colorectal cancer tissue microarray

Human colorectal cancer tissue microarrays were obtained from Shanghai Outdo Biotech CO., LTD. (HCol-Ade180Sur-06, Shanghai, China) containing 86 samples of different stages of colorectal cancers.

### Plasmid constructions and siRNAs

The 8 × Gli-binding site luciferase reporter plasmid (GliBS) and mutant 8 × Gli-binding site luciferase reporter (mGliBS) were kindly provided by Dr. Sasaki. The following plasmids were obtained from Addgene: pCDNA3 Flag MKK7B2Jnk1a1(APF) (#19730, Addgene), pCDNA3 Flag MKK7B2Jnk1a1 (#19726, Addgene), pCDNA3 Flag Jnk1a1(apf) (#13846, Addgene), pCDNA3 Jnk1a1 (#13798, Addgene), pRK5-HA-Ubiquitin-WT (#17608, Addgene), pBShh (#13996, Addgene). To generate the expression constructs for Gli2, the PCS2-MT GLI2 FL vector obtained from Addgene (#17648, Addgene) was subcloned into either pcDNA3.1 Myc or pcDNA3.1 HA vector. The Myc tagged Gli2-expressing plasmid was further used to generate its mutants Gli2 T1546A, and Gli2 T1546E using the Quick Change multi site-directed mutagenesis kit from Stratagene (La Jolla, CA). The fragment 1 of Gli2 (Gli2-F1) including 160–600 amino acids, the Gli2-F2 including 500–1100 amino acids, and the fragment Gli2-F3 including 1000–1587 amino acids were acquired through PCR and inserted in pGEX-6P-1 (GE Healthcare) for constructing His fusion protein. The site mutant of Gli2-F3 T1546A was generated from pGEX-6P-1 Gli2-F3 by using the Quick Change multisite-directed mutagenesis kit from Stratagene.

The pre-designed siRNAs targeting Gli2 and Smo were obtained from GenePharma (Shanghai, China).

### Dual-luciferase reporter assay

Cells were seeded in 24-well plate and transfected with GliBS or mGliBS, together with CMV/Renilla vector serving as an internal standard. After overnight, the cells were subjected to various treatments as indicated, followed by luciferase assay using a dual luciferase reporter assay system (#E1960, Promega) according to the manufacturer’s instruction.

### Real-time PCR analysis

Total RNA was extracted from cells using an RNAiso Plus Kit (TaKaRa; Dalian, China) according to the manufacturer’s instructions, and directly processed to cDNA by reverse transcription with a SuperScript III Kit (#RR036A, TaKaRa). Semiquantitative PCR amplification was conducted using Stratagene mx3005p (Agilent). The quantitative PCR amplifications were performed in triplicate with a SYBR Green Kit (#RR086A, TaKaRa) in an iCycler iQ system (Bio-Rad; Hercules, CA). The mRNA expression levels of the interested genes were normalized to that of *Gusb*.

### Cell proliferation assay

Cells were plated in 96-well plate. After various treatments as indicated, the cells were subjected to proliferation assays using a BrdU Cell Proliferation Assay Kit (#2750, Millipore) following the manufacturer’s protocol.

### Immunoprecipitation, in vitro kinase assay, and immunoblot analysis

Cell lysis was obtained with lysis buffer (50 mM Tris pH 8.0, 150 mM NaCl, 10% glycerol, 1 mM EDTA, 50 mM NaF, and 0.1% NP-40) supplemented with protease and phosphatase inhibitors. For immunoprecipitation, soluble products were incubated with antibodies as indicated at 4 °C overnight, followed by incubation with Protein A/G Plus-Agarose (Santa Cruz Biotechnology) at 4 °C for 2 h. Complexes were separated from the beads and then boiled for 10 min. For in vitro kinase assay, purified His-tagged Gli2 fragment proteins were incubated with purified JNK1 proteins (Millipore) in the presence of 50 mM ATP in a kinase buffer [20 mM MgCl_2_,100 mM ATP, 2 mM DTT, 50 mM inhibitors as indicated and 40 mM Tris-HCl(pH 7.5)] for 30 min at 30 °C. The kinase reaction was quenched by the addition of 5× sample loading buffer and boiling at 100 °C for 10 min. Reaction products were subjected to sodium dodecyl sulfate polyacrylamide gel electrophoresis (SDS-PAGE) and Coomassie Blue staining.

The proteins were separated by SDS-PAGE, electrophoretically transferred to polyvinylidene fluoride membranes, and subjected to routine immunoblot analysis with interested antibodies as indicated. The antibody against the phosphorylation of Gli2T1546 was produced using the synthetic phosphorylated peptides CSSRLTT(p)PRN as antigen, and purified on a phosphopeptide column (EZBiolab Inc, Carmel, IN).

### Mass spectrometry and data analysis

Recombinant His-Gli2 fragments purified from *E. coli* was incubated in vitro with or without recombinant JNK1 protein in the presence of 50 mM ATP at 30 °C for 30 min. The samples were resolved by SDS-PAGE and stained with Coomassie blue. The Gli2 fragment bands were cut and digested. The resulting peptides were subjected to the enrichment of phosphorylated peptides by using TiO_2_. The enriched phosphorylated peptides were analyzed on the Q Exactive HF mass spectrometer (Thermo Scientific). The identification and quantification of phosphorylated peptides was done by MaxQuant. The tandem mass spectra were searched against UniProt human protein database together with a set of commonly observed contaminants. The precursor mass tolerance was set as 20 ppm, and the fragment mass tolerance was set as 0.1 Da. The cysteine carbamidomethylation was set as a static modification, and the methionine oxidation as well as serine, threonine and tyrosine phosphorylations were set as variable modifications. The FDR at peptide spectrum match level and protein level were controlled below 1%.

### Immunohistochemistry

Hematoxylin and eosin staining was routinely performed. For immunohistochemistry, tissue microarray slides were deparaffinised, rehydrated by an alcohol series, and incubated with citrate buffer at 95 °C for 40 min for antigen retrieval and incubated overnight at 4 °C with the primary antibodies as indicated. After three washes, tissue microarray slides were incubated with biotinylated anti-mouse or anti-rabbit IgG and incubated with avidin-biotin-peroxidise conjugates. For the quantitative analysis, a Histo score (*H*-score) was calculated based on the staining intensity and percentage of stained cells using the Aperio ScanScope systems. The intensity score was defined as follows: 0, no appreciable staining in cells; 1, weak staining in cells comparable with stromal cells; 2, intermediate staining; 3, strong staining. The fraction of positive cells was scored as 0–100%. The *H*-score was calculated by multiplying the intensity score and the fraction score, producing a total range of 0–300. Tissue sections were examined and scored separately by two independent investigators blinded to the clinicopathologic data.

### Statistical analysis

Statistical differences were analyzed by the two-tailed Student’s *t*-test, Spearman’s test, and Chi-square test. *P* value less than 0.05 was considered as significant. Asterisks denote statistical significance (^#^*P* > 0.05; *P < 0.05; **P < 0.01; and ***P < 0.001).

## Results

### PGE2 non-canonically promotes the Hh activity in colorectal cancer cells in a Smo-independent manner

**Fig. 1:**
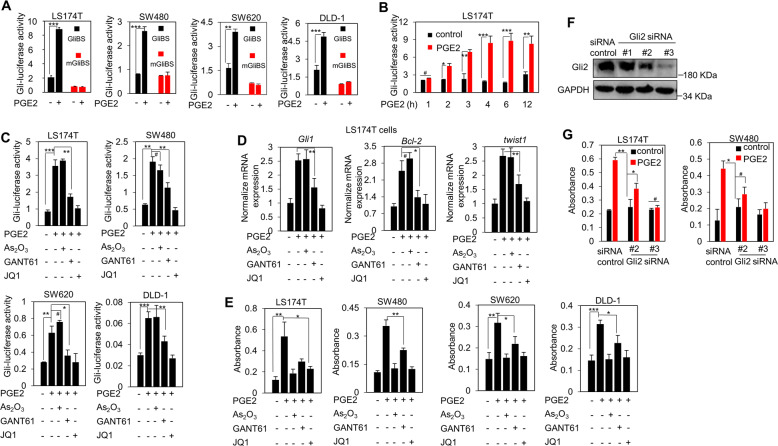
PGE2 non-canonically promotes the Hh pathway activity in colorectal cancer cells. **A**, **B** Luciferase assay for Gli transcriptional activity in colorectal cancer cells with or without PGE2 (1 μM) stimulation. Cells were transfected with GliBS-Luciferase or mGliBS-Luciferase plasmids plus TK-Renilla, and then treated with PGE2 for 6 h (**A**) or 1–12 h (**B**). GliBS is a Gli-responsive reporter, and mGliBS is a Gli-unresponsive reporter. Error bars represent SD (*n* = 3). **C** Luciferase assay for Gli transcriptional activity in colorectal cancer cells subjected to PGE2 with or without Gli inhibitors As_2_O_3_ (10 μM), GANT61 (20 μM), and JQ1 (1 μM) for 6 h. Error bars represent SD (*n* = 3). **D** The mRNA levels of Gli target genes in colorectal cancer cells were examined by real-time PCR and normalized to the mRNA level of *gusb*. Cells were exposed to PGE2 (1 μM) with or without As_2_O_3_ (10 μM), GANT61 (20 μM), and JQ1 (1 μM) for 6 h. Error bars represent SD (*n* = 3). **E** The proliferation of colorectal cancer cells was examined with BrdU assays. Cells were exposed to PGE2 (1 μM) with or without As_2_O_3_ (10 μM), GANT61 (20 μM), and JQ1 (1 μM). Error bars represent SD (n = 3). **F** Immunoblot analysis of Gli2 expression in LS174T cells transfected with Gli2 siRNA. **G** The proliferation of colorectal cancer cells LS174T and SW480 was examined with BrdU assays. The cells were transfected with Gli2 siRNA followed by treatment with or without PGE2 (1 μM). Error bars represent SD (*n* = 3).

Chronic inflammation has emerged as one of the key risk factors for initiation, and progression of colorectal cancers. Using Gli-dependent luciferase reporter activity as a Hh activity readout, we observed that PGE2 profoundly increased the Gli activity in multiple colorectal cancer cell lines, including LS174T, SW480, SW620, and DLD-1 cells (Fig. 1A), in a time-dependent manner, reaching maximum at about 6 h (Fig. 1B). However, PGE2 exhibited no effect on the luciferase activity of the mutant Gli-dependent luciferase reporter plasmid (Fig. 1A). PGE2-stimulated Gli activity could be obviously abolished by specific Gli antagonists GANT61 [23], and JQ1 [24], but not by As_2_O_3_ [25, 26] (Fig. 1C), possibly due to the context-dependent characteristics of Gli inhibitors [27]. These observations were recapitulated by using Gli target genes *Gli1* [28], *Bcl-2* [29], and *twist1* [30] as readouts of Hh activity in LS174T cells (Fig. 1D). Comparable to the elevated Gli activity, PGE2 increased the proliferation of all of these tested colorectal cancers cells in a Gli-dependent manner, as revealed by the inhibitory effect of either Gli inhibitors GANT61 and JQ1 (Fig. 1E), or genetic silencing Gli2 by small interfering RNA (siRNA) (Fig. 1F, G). Notably, the proliferation of colorectal cancers cells blocked by As_2_O_3_ might be caused by its pro-apoptotic effect [31, 32] (Fig. 1E), rather by influence on the Hh activity, due to its inability of inhibiting Hh activity (Fig. 1C, D). Taken together, these results demonstrate that PGE2 may non-canonically promote Hh activity and consequently the proliferation of colorectal cancer cells.

In addition to PGE2, numerous inflammatory cytokines have also been shown to be involved in initiation, and progression of colorectal cancers, such as tumor necrosis factor α (TNF-α), and interleukin 6 (IL-6) [33]. We then asked whether TNF-α, and IL-6 can also non-canonically promote Hh activity. Unexpectedly, we observed that both TNF-α, and IL-6 exhibited no significant effect on the Gli-luciferase activity (Fig. S1A). Consistently, both TNF-α, and IL-6 failed to increase the mRNA expression of Gli target genes *Gli1*, *Bcl-2*, and *twist1* (Fig. S1B). These data suggest that PGE2 may be the predominant inflammatory factor involved the abnormal and non-canonical activation of Hh activity during the initiation, and progression of colorectal cancer.

Considering that Smo is a critical molecular target of the vast majority of current Hh inhibitors [34, 35], we then set out to explore whether Smo is involved in the PGE2-provoked Hh activity and proliferation of colorectal cancer cells. We observed that in contrast to Gli inhibitors JQ1, either Smo antagonists GDC-0449 (GDC) [36], and NVP-LDE-225 (LDE) [37] (Fig. S2A, B), or genetic silencing Smo by siRNA (Fig. S2D–F) was unable to block the Hh activity in response to PGE2, as evidenced by no alterations of Gli-luciferase activity (Fig. S2A and S2E) and mRNA expression of Hh target genes *Gli1*, *Bcl-2*, and *twist1* (Fig. S2B–F). Consistent with the unaffected Hh activity, either Smo antagonists GDC, and LDE (Fig. S2C), or genetic silencing Smo (Fig. S2G) also failed to inhibit the proliferation of colorectal cancer cells stimulated by PGE2. These data suggest that Smo is dispensable for PGE2-stimulated Gli activity and proliferation of colorectal cancer cells. Meanwhile, we also observed that N-terminal SHh conditioned medium (ShhN CM) failed to stimulate the Gli activity and proliferation of colorectal cancer cells (Fig. S2A–C), consistent with the observations in previous study [11].

### PGE2 protects Gli2 from ubiquitin-proteasomal degradation by activating JNK

**Fig. 2:**
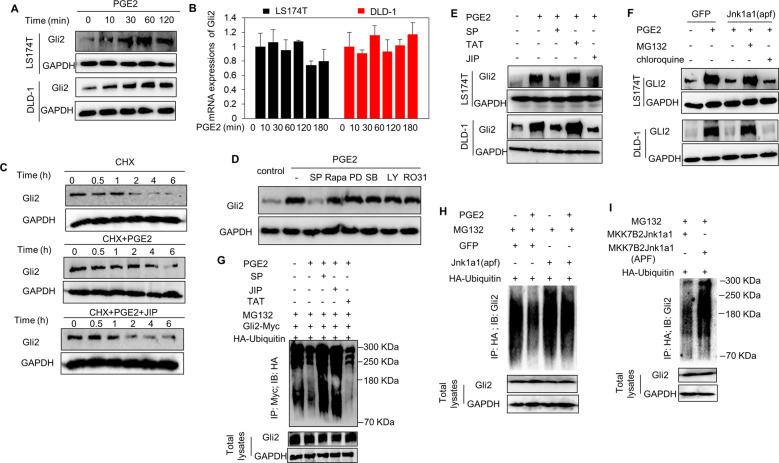
PGE2 protects Gli2 from ubiquitin-proteasomal degradation by activating JNK. **A** Expression of Gli2 protein in LS174T and DLD-1 cells treated with PGE2 for different time intervals as indicated was analyzed by western blotting. **B** The mRNA level of Gli2 in LS174T and DLD-1 cells treated with PGE2 for different time intervals was examined by real-time PCR and normalized to the mRNA level of *gusb*. Error bars represent SD (*n* = 3). **C** Expression of Gli2 protein in LS174T cells treated with CHX (10 μg/ml), PGE2 (1 μM), and JIP (2 μM) as indicated for different time intervals was analyzed by western blotting. **D** Immunoblot analysis of Gli2 protein expression in LS174T cells treated with PGE2 (1 μM) in combination with SP (10 μM), Rapa (100 nM), PD (10 μM), SB (10 μM), LY (10 μM), RO31 (1 μM) for 1 h, respectively. **E** Immunoblot analysis of Gli2 protein expression in LS174T and DLD-1 cells treated with PGE2 with or without SP, TAT, and JIP as indicated for 1 h. **F** Immunoblot analysis of Gli2 protein expression in LS174T and DLD-1 cells. Cells were transiently transfected with GFP or Jnk1a1(apf), followed by exposure to PGE2 with or without MG132 (10 μM), and chloroquine (10 μM) as indicated for 1 h. **G** Immunoprecipitation-western blot analysis of the ubiquitination of Gli2 (detected with anti-HA) in LS174T cells expressing HA-tagged ubiquitin, and Myc-tagged Gli2 with various combinations of treatment with PGE2, SP (10 μM), JIP (2 μM), TAT (2 μM), and MG132 (10 μM) as indicated 1 h. **H** Immunoprecipitation-western blot analysis of the ubiquitination of Gli2 (detected with anti-Gli2) in LS174T cells expressing GFP, Jnk1a1(apf), and HA-tagged ubiquitin with various combinations of treatment with PGE2, and MG132 as indicated for 1 h. **I** Immunoprecipitation-western blot analysis of the ubiquitination of Gli2 (detected with anti-Gli2) in LS174T cells expressing MKK7B2Jnk1a1, MKK7B2Jnk1a1 (APF), and HA-tagged ubiquitin after exposure to MG132 as indicated for 1 h.

Given that ubiquitin proteasomal degradation is one of the major means for canonical Hh pathway to regulate Gli activity [3], we then asked whether PGE2 also promotes the Gli activity via ubiquitin proteasomal degradation. PGE2 treatment of colorectal cancer cells increased the abundance of Gli1 (Fig. S3A) and Gli2 proteins (Fig. 2A), reaching maximum at approximately 1 h, with no appreciable effect on the expression of Gli3 protein (Fig. S3A). As Gli2 is regarded as the primary activator for initiating the Hh pathway [3], we thus focused our further investigations on Gli2. Despite the increase of Gli2 abundance at protein level by PGE2, the transcript level of Gli2 was not altered by PGE2 treatment (Fig. 2B). Moreover, in the presence of de novo protein synthesis inhibitor cycloheximide (CHX), PGE2 obviously reduced the turnover of Gli2 in LS174T cells (Fig. 2C and Fig. S3B). These observations collectively indicate that PGE2 regulates Gli2 protein expression by increasing its protein stability at post-translational level. Considering that kinases have been the most successful targets for developing mechanisms-based anticancer drugs, we continued to determine whether kinases are involved in PGE2-induced Gli2 protein expression at posttranslational level. Among multiple small molecular inhibitors of kinases, including SP600125 (SP), Rapamycin (Rap), PD98059 (PD), SB203580 (SB), LY294002 (LY), and Ro31-8220 (Ro31), only JNK inhibitor SP was able to abrogate PGE2-elicited enhancement of Gli2 expression (Fig. 2D). Meanwhile, we found that PGE2 treatment resulted in phosphorylation of JNK, reaching maximum at around 10–30 min (Fig. S3C). These observations indicate the involvement of JNK in the posttranslational modification of Gli2. Such argument was further supported by the observations of restoring PGE2-elicited decrease of Gli2 turnover by peptide JNK inhibitor TAT-TI-JIP (JIP) (Fig. 2C and Fig. S3B) and decreasing Gli2 expression by JIP and SP (Fig. 2E). TAT was used as a negative control of peptide JNK inhibitor JIP. Meanwhile, we found that a proteasomal inhibitor MG132, but not a lysosomal inhibitor chloroquine, obviously rescued the down-regulation of Gli2 expression elicited by JNK dominant negative mutant Jnk1a1(apf) [38] (Fig. 2F), suggesting that PGE2 increased the stability of Gli2 by protecting Gli2 from proteasomal degradation via activating JNK. Furthermore, PGE2 treatment obviously diminished the ubiquitination of Gli2, which could be restored by small molecular JNK inhibitors SP and JIP (Fig. 2G) or Jnk1a1(apf) (Fig. 2H). Meanwhile, we observed that JNK activation by transfection of MKK7B2Jnk1a1, a constitutively active JNK mutant [38], significantly abolished the ubiquitination of Gli2 (Fig. 2I). Taken together,these data demonstrate that colorectal cancer cells may hijack PGE2 to enable the evasion of Gli2 from ubiquitin proteasomal degradation by activating JNK.

### JNK activation by PGE2 protects Gli2 from ubiquitin-proteasomal degradation by phosphorylating Gli2 at Thr1546

**Fig. 3:**
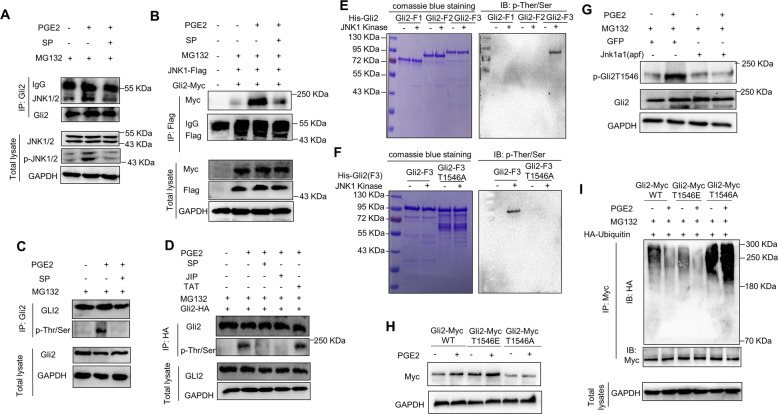
JNK activation by PGE2 protects Gli2 from ubiquitin-proteasomal degradation by phosphorylating Gli2 at Thr1546. **A** Immunoprecipitation-western blot analysis of the interaction between endogenous Gli2 with JNK in 293T cells treated with PGE2, SP, and MG132 as indicated for 1 h. **B** Immunoprecipitation-western blot analysis of the interaction between exogenous Gli2 with JNK in 293T cells expressing Flag-tagged JNK1, andMyc-tagged Gli2 after exposure to PGE2, SP, and MG132 as indicated for 1 h. **C** Immunoprecipitation-western blot analysis of Thr/Ser phosphorylation of endogenous Gli2 in LS174T cells after exposure to PGE2, SP, and MG132 as indicated for 1 h. **D** Immunoprecipitation-western blot analysis of Thr/Ser phosphorylation of exogenous Gli2 in LS174T cells expressing HA-tagged Gli2 after exposure to PGE2, SP, and MG132 as indicated for 1 h. **E** The in vitro kinase assay was performed with recombinant JNK1 and indicated His-tagged Gli2 fragments, followed by immunoblotting with anti-phosphorylation-Thr/Ser antibody. **F** The in vitro kinase assay was performed with recombinant JNK1 and His-tagged Gli2-F3 fragments and His-tagged Gli2-F3 harboring the alanine substitution of theronine 1546 (Gli2-F3 T1546A), followed by immunoblotting with anti-phosphorylation-Thr/Ser antibody. **G** Immunoblot analysis of Thr1546 phosphorylation of Gli2 in LS174T cells expressing GFP, and Jnk1a1(apf) after exposure to PGE2, MG132 as indicated for 1 h using specific phosphor-Gli2Thr1546 antibody. **H** Immunoblot analysis of Myc-tagged Gli2 (Gli2-Myc WT), Myc-tagged Gli2 with the glutamine substitution of threonine 1546 (Gli2-Myc T1546E), and Myc-tagged Gli2 with the alanine substitution of threonine 1546 (Gli2-Myc T1546A) in LS174T cells treated with PGE2 as indicated for 1 h. **I** Immunoprecipitation-western blot analysis of the ubiquitination of Gli2 (detected with anti-HA) in LS174T cells expressing HA-tagged ubiquitin, Gli2-Myc WT, Gli2-Myc T1546E, and Gli2-Myc T1546A after exposure to PGE2, and MG132 as indicated for 1 h.

Given that JNK is a Serine/Threonine (Ser/Thr) kinase, we then set out to explore whether JNK enables the evasion of Gli2 from ubiquitin proteasomal degradation by phosphorylating Gli2. We first characterized the physical interaction between JNK and Gli2. As shown in Fig. 3A, PGE2 promoted the binding of endogenous JNK1 to endogenous Gli2, and this physical interaction was dramatically diminished by JNK inhibitor SP (Fig. 3A). This phenomenon was recapitulated with exogenous JNK1 and Gli2 (Fig. 3B). Meanwhile, by immunoblotting with an anti-phospho-Ser/Thr antibody, we observed that PGE2 treatment caused Ser/Thr phosphorylation of endogenous (Fig. 3C) and exogenous (Fig. 3D) Gli2, and JNK inhibitor JIP and SP obviously abolished the PGE2-elicited Ser/Thr phosphorylation of Gli2 (Fig. 3C, D). These results indicate that Gli2 represents a substrate of JNK.

Furthermore, in vitro kinase assay showed that a Gli2 fragment containing the 1000–1587 amino acids, Gli2-F3, was phosphorylated by recombinant JNK1 (Fig. 3E), confirming that Gli2 is a bona fide substrate of the kinase JNK. To further identify the JNK-mediated phosphorylation sites on Gli2-F3, we harvested the Gli2-F3 protein from in vitro kinase assay and performed mass spectrometry analysis. The mass spectrometry analysis detected the phosphorylation of Gli2-F3 on Thr1546 (Fig. S4A). Furthermore, mutation of Thr1546 to alanine (Gli2-F3 T1546A) abolished the phosphorylation in response to recombinant JNK1 (Fig. 3F), confirming that Thr1546 is the phosphorylation site of Gli2 protein by JNK1.

To further explore whether Gli2 may be phosphorylated by PGE2-JNK signaling axis at Thr1546, we developed a rabbit polyclonal antibody that specifically recognizes phosphorylated Thr1546 of Gli2 (p-Gli2T1546). Using this antibody, we observed that PGE2 treatment resulted in obvious Gli2 Thr1546 phosphorylation, which was diminished by limiting Gli2 expression with Gli2 siRNA (Fig. S4B), therefore, confirming the suitability of this antibody. Ectopic expression of MKK7B2Jnk1a1 [38], but not its negative control MKK7B2Jnk1a1(APF) [38], led to exogenous Gli2 Thr1546 phosphorylation (Fig. S4C). Furthermore, suppressing the JNK function by Jnk1a1(apf) [38] (Fig. 3G) or JNK inhibitor SP and JIP (Fig. S4D) significantly abolished the Gli2 Thr1546 phosphorylation in response to PGE2 (Fig. 3G and Fig. S4D). These results further strengthen that JNK after activated by PGE2 may phosphorylate Gli2 at Thr1546.

To further elucidate the molecular basics behind how JNK regulates the ubiquitination of Gli2, we then continued to examine whether Gli2 Thr1546 phosphorylation is involved in JNK-mediated evasion of Gli2 from proteasomal-ubiquitin degradation in response to PGE2. We found that mutation of Thr1546 to glutamine (Gli2T1546E), which is structurely similar with phosphorylation amino acid to mimick the phoshphorylated Gli2, rendered exogenous Gli2 resistant to degradation in the absence of PGE2 (Fig. 3H). Conversely, PGE2 lost the ability to protect exogenous Gli2 from degradation when Thr1546 was mutated to alanine (Gli2T1546A), which was designed to mimic the non-phosphorylated Gli2 (Fig. 3H). Furthermore, as shown in Fig. 3I, exogenous Gli2T1546E exhibited much less ubiquitination level in the absence of PGE2 when compared to that of Gli2 wild type (Gli2 WT). However, Gli2T1546A possessed much more ubiquitination level no matter whether PGE2 exposure or not (Fig. 3I). These data demonstrate that the evasion of Gli2 from proteasomal-ubiquitin degradation in response to PGE2 treatment requires JNK activation, thereby further phosphorylating Gli2 at Thr1546.

### JNK is required for the Hh activation and proliferation of colorectal cancer cells elicited by PGE2

**Fig. 4:**
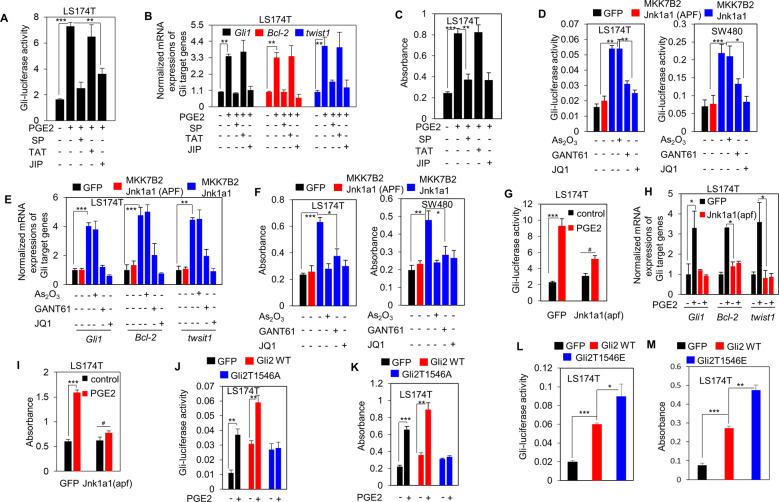
JNK is required for the Hh activation and proliferation of colorectal cancer cells elicited by PGE2. **A** Luciferase assay for Gli transcriptional activity in colorectal cancer cells LS174T treated by PGE2, SP, TAT, JIP as indicated for 6 h. Error bars represent SD (*n* = 3). **B** The mRNA levels of Gli target genes in LS174T cells treated by PGE2, SP, TAT, JIP as indicated for 6 h were examined by real-time PCR and normalized to the mRNA level of *gusb*. Error bars represent SD (*n* = 3). **C** The proliferation of colorectal cancer cells LS174T treated by PGE2, SP,TAT, JIP as indicated for 6 h was examined with BrdU assays. Error bars represent SD (*n* = 3). **D** Luciferase assay for Gli transcriptional activity in colorectal cancer cells LS174T and SW480 cells expressing GFP, MKK7B2Jnk1a1, and MKK7B2Jnk1a1 (APF) after exposure to Gli inhibitors As_2_O_3_ (10 μM), GANT61 (20 μM), and JQ1 (1 μM) as indicated for 6 h. Error bars represent SD (*n* = 3). **E** The mRNA levels of Gli target genes in LS174T cells expressing GFP, MKK7B2Jnk1a1, and MKK7B2Jnk1a1 (APF) were examined by real-time PCR and normalized to the mRNA level of *gusb* after exposure to Gli inhibitors As_2_O_3_ (10 μM), GANT61 (20 μM), and JQ1 (1 μM) as indicated for 6 h. Error bars represent SD (*n* = 3). **F** The proliferation of colorectal cancer cells LS174T and SW480 cells expressing GFP, MKK7B2Jnk1a1, and MKK7B2Jnk1a1 (APF) was examined with BrdU assays after exposure to Gli inhibitors As_2_O_3_ (10 μM), GANT61 (20 μM), and JQ1 (1 μM) as indicated for 6 h. Error bars represent SD (*n* = 3). **G** Luciferase assay for Gli transcriptional activity in colorectal cancer cells LS174T expressing GFP, and Jnk1a1 (APF) after treated by PGE2 for 6 h. Error bars represent SD (*n* = 3). **H** The mRNA levels of Gli target genes in colorectal cancer cells LS174T expressing GFP, and Jnk1a1 (APF) after treated by PGE2 for 6 h were examined by real-time PCR and normalized to the mRNA level of gusb. Error bars represent SD (*n* = 3). **I** The proliferation of colorectal cancer cells LS174T expressing GFP, and Jnk1a1 (APF) after treated by PGE2 for 6 h was examined with BrdU assays. Error bars represent SD (*n* = 3). **J** Luciferase assay for Gli transcriptional activity in colorectal cancer cells LS174T cells expressing GFP, Gli2 WT, and Gli2T1546A after exposure to PGE2 as indicated for 6 h. Error bars represent SD (*n* = 3). **K** The proliferation of colorectal cancer cells LS174T cells expressing GFP, Gli2 WT, and Gli2T1546A after exposure to PGE2 was examined with BrdU assays. Error bars represent SD (*n* = 3). **L** Luciferase assay for Gli transcriptional activity in colorectal cancer cellsLS174T cells expressing GFP, Gli2 WT, and Gli2T1546E. Error bars represent SD (*n* = 3). **M** The proliferation of colorectal cancer cells LS174T cells expressing GFP, Gli2 WT, and Gli2T1546E was examined with BrdU assays. Error bars represent SD (*n* = 3).

As we have determined that JNK is required for the evasion of Gli2 from proeasomal-ubiquitin degradation in response to PGE2 by phosphorylating Gli2 at Thr1546, we then set out to test whether JNK is involved in the Hh activation and the consequent proliferation of colorectal cancer cells elicited by PGE2. We observed that JNK inhibition by JIP and SP abrogated the enhancement of both Gli luciferse reporter activity (Fig. 4A) and transcripts of Gli target genes *Gli1*, *Bcl-2*, and *twist1* (Fig. 4B) in response to PGE2. The decrease of Hh activity elicited by SP and JIP paralleled their inhibitory effect on proliferation of colorectal cancer cells (Fig. 4C). Moreover, artificially forced expression of JNK constitutively active mutant MKK7B2Jnk1a1 dramatically increased the Hh activity, as reflected by Gli-luciferase activity (Fig. 4D) and transcripts of Gli target genes *Gli1*, *Blc-2*, and *twist1* (Fig. 4E), which both were obviously diminished by specific molecular Gli antagonists GANT61, and JQ1, but not by As_2_O_3_ (Fig. 4D, E). Comparable results were obtained when we measured the proliferation of colorectal cancer cells (Fig. 4F). Similar to above data as shown in Fig. 1, As_2_O_3_ exhibited no inhibitory effect on the Hh activity provoked by MKK7B2Jnk1a1, but suppressed the MKK7B2Jnk1a1-stimulated proliferation of colorectal cancer cells (Fig. 4D–F). These observations were further confirmed by ectopic expression of JNK dominant negative mutant Jnk1a1(apf) (Fig. 4G–I). Hence, these data suggest that JNK activated by PGE2 is essential for PGE2-elicited Hh activity and the consequent proliferation of colorectal cancer cells.

We next continued to explore whether JNK-mediated Gli2 Thr1546 phosphorylation is essential for Hh activity and proliferation of colorectal cancer cells in response to PGE2. Contrary to transfection with GFP, and Gli2 WT, transfection with Gli2T1546A rendered PGE2 unable to promote the Gli-luciferase activity and proliferation of LS174T cells (Fig. 4J, K). On the other hand, transfection of Gli2T1546E obviously increased the Gli-luciferase activity and proliferation of LS174T cells (Fig. 4L, M). Hence, these results showed that JNK-mediated Gli2 Thr1546 phosphorylation is required for the PGE2-dependent Hh activity and proliferation of colorectal cancer cells.

### PGE2-JNK signaling axis promotes intestinal tumor growth by non-canonically stimulating Hh activity in a Smo-independent manner in *Apc*^Min/+^ mice

**Fig. 5:**
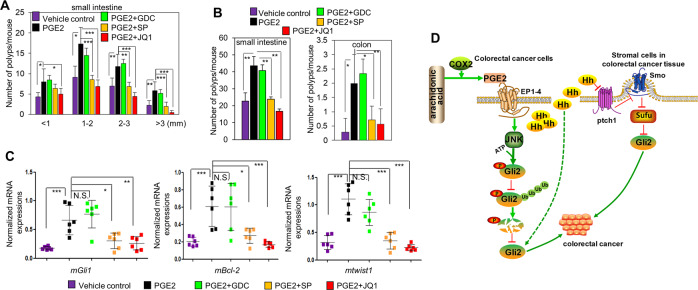
PGE2-JNK signaling axis promotes intestinal tumor growth by non-canonically stimulating Hh activity in a Smo-independent manner in *Apc*^Min/+^ mice. **A**, **B** The intestinal polyp number and size in *Apc*^Min/+^ mice treated with PGE2,GDC,SP, and JQ1 as indicated for 2 months. Error bars represent SD (*n* = 6). **C** The mRNA levels of Gli target genes in small intestine of mice treated with PGE2, GDC, SP, and JQ1 as indicated for 2 months. Error bars represent SD (*n* = 6). **D** Schematic model depicting the activation of Hh signaling pathway in cancer cells and stromal cells in colorectal cancer.

We next set out to confirm whether PGE2-JNK signaling axis may promote the Hh activity in vivo using the *Apc*^Min/+^ mice, which has been frequently used as a model for human familial adenomatous polyposis and a premalignant model for human colorectal cancer [39, 40]. Treatment of *Apc*^Min/+^ mice with PGE2 accelerated intestinal adenoma growth, which was obviously suppressed by JNK and Gli inhibitor SP and JQ1, respectively, but not by Smo inhibitor GDC (Fig. 5A, B). In accordance with the observations on tumor burden, PGE2 treatment resulted in dramatic increase of Hh activity as reflected by the *Gli1*, *Bcl-2*, and *twist1* mRNA expression of the intestinal adenoma cells isolated from the *Apc*^Min/+^ mice (Fig. 5C). Furthermore, we observed that small molecular inhibitors SP and JQ1, significantly abolished the enhancement of Hh activity caused by PGE2 treatment, as evidenced by the downregulation of *Gli1*, *Bcl-2*, and *twist1* mRNA expression (Fig. 5C). Meanwhile, Smo inhibitor GDC exhibited no effect on the Hh activity provoked by PGE2 (Fig. 5C). Collectively, these data obtained from *Apc*^Min/+^ mice further confirmed that PGE2-JNK signaling axis may non-canonically promote Hh activity in a Smo-independent manner by protecting Gli2 from degradation (Fig. 5D), and consequently promote the development of colorectal cancer growth.

### Phosphorylated JNK and Gli2T1546 are correlated with Gli2 expression in colorectal cancer

**Fig. 6:**
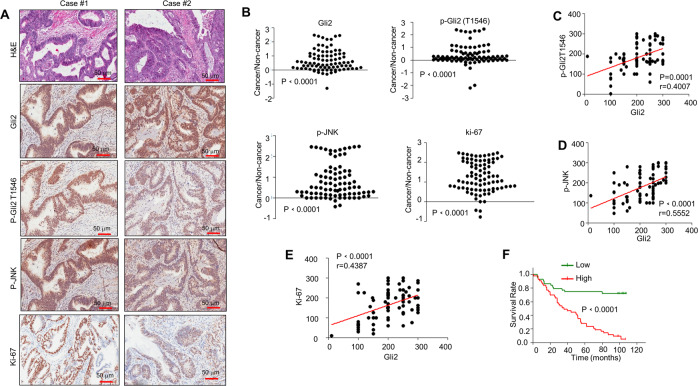
Phosphorylated JNK and Gli2T1546 are correlated with Gli2 expression in colorectal cancer. **A** H&E staining and IHC staining of the primary human CRC tissue microarray. Scale bar, 50 μm. **B** Scatter plot showing the Gli2, p-Gli2T1546, p-JNK, and ki-67 staining level in individual tumors as a ratio of staining in CRC versus paired non-cancer tissue. *n* = 86. **C** Correlation plot of p-Gli2T1546 and Gli2 IHC staining (arbitrary units). Correlation was evaluated by nonparametric Spearman test. The coefficient of correlation (*r*), and the *p* value (*p*) are indicated. *n* = 86. **D** Correlation plot of p-JNK and Gli2 IHC staining (arbitrary units). Correlation was evaluated by nonparametric Spearman test. The coefficient of correlation (*r*), and the *p* value (*p*) are indicated. *n* = 86. **E** Correlation plot of ki-67 and Gli2 IHC staining (arbitrary units). Correlation was evaluated by nonparametric Spearman test. The coefficient of correlation (*r*), and the *p* value (*p*) are indicated. *n* = 86. **F** Kaplan–Meier survival analysis of CRC cases separated into two groups by the median value for phospho-Gli2^T1546^ staining. The *p* value was calculated by log rank test.

To determine the pathological relevance of our findings, we analyzed the relationship between the expression of Gli2 and the phosphorylation of JNK. All the commercially available antibodies used in this experiment were suitable for IHC as indicated by the vendor. The suitability of p-Gli2 (T1546) for immunohistochemistry (IHC) staining was verified using phospho-peptide and non-phospho-peptide, as shown in Fig. S5. Examination of the tissue array of human colorectal cancer and adjacent non-tumor tissues with IHC staining revealed the increased expression of Gli2, p-JNK, p-Gli2T1546, and ki-67 compared with those in adjacent non-tumor tissues (Fig. 6A, B). Meanwhile, the expression of Gli2 was associated with expression of p-Gli2T1546, p-JNK, and ki-67, respectively (Fig. 6C, D). Furthermore, Kaplan–Meier survival analysis showed that the survival time for colorectal cancer patients with high expression of p-Gli2T1546 was significantly shorter than those with low expression of p-Gli2T1546 (Fig. 6E). Take together, those data suggest that there is close association between the expression of these proteins and the colorectal cancer development.

## Discussion

Ligand-independent aberrant activation of Hh pathway caused by genetic alteration of its critical components has been well documented to be critical for driving the initiation and progression of basal cellular carcinoma, as well as subsets of medellublastoma [5]. However, the contribution of Hh pathway to the majority of solid tumors, such as pancreas, prostate, and colorectal cancers, which have been supposed to possess aberrant activation of Hh in a manner of ligand-dependent autocrine or paracrine activation, remains far from being fully elucidated [41]. As for sporadic colorectal cancer, it has been initially shown that ligand-dependent autocrine activation of Hh pathway in colon cancer cells is required for the growth and metastasis of colorectal cancers [8, 9]. Meanwhile, accumulating evidence suggests that ligand-dependent paracrine activation of Hh pathway in tumor stromal cells provides a supportive microenvironment for the initiation and progression of colorectal cancers [10–14]. However, Gerling et al. recently reported that stromal Hh signaling was found to be downregulated and promoted the development of colorectal cancer, while its restoration might function as a colonic tumor suppressor [16].

Given the complexity of the initiation and progression of colorectal cancer, we explored the impact of Hh signaling to the proliferation of colorectal cancer cells under the circumstance of inflammatory factors intimately involved the initiation and progression of colorectal cancer. We demonstrate that PGE2 non-canonically promotes Hh activation and consequently the proliferation of colorectal cancer cells in a Smo-independent manner. Meanwhile, we found that PGE2 might be the predominant inflammatory factor involved in provoking the non-canonical activation of Hh in colorectal cancer cells, as other inflammatory factor intimately associated with colorectal cancer, such as IL-6, and TNF-α, failed to enhance the Hh activity in colorectal caner cells. Mechanistically, PGE2 activates the downstream effectors JNK, thereby protecting Gli2 from proteasomal-ubiquitin degradation by phosphorylating Gli2 at Thr1546. Wang et al. demonstrated that mTOR/S6K1 pathway non-canonically stimulated Gli activity through S6K1-mediated Gli1 phosphorylation at Ser84, which releases Gli1 from its endogenous inhibitor, SuFu protein, in esophageal adenocarcinoma cells [42]. However, our lab found that colorectal cancer cells, such as LS174T and SW480 cells exhibited little appreciable expression of Sufu protein (data to be published), suggesting a dictinct mechanisms for kinase JNK’s effect on Gli activity. Hence, our study provides an novel insight into the mechanisms underlying how Hh signaling is involved in the initiation and progression of colorectal cancer, and a rationale for the future evaluation of chemopreventive and selectively therapeutic strategies for colorectal cancers by targeting PGE2-JNK-Gli signaling route.

Our present study, together with reports from other labs, indicates that Hh activity in colorectal cancer tissues is stimulated by two distinct manners: (i) canonical and Smo-dependent activation of Hh activity in colorectal cancer cells and stromal cells [8–14], and (ii) non-canonical and Smo-independent activation provoked by PGE2 in colorectal cancer cells (Fig. 5D). This may possibly explain the disappointing clinical trial results of Smo inhibitors for treatment of colorectal cancer [15], as Smo inhibitors can merely abolish the canonical and Smo-dependent Hh activity, while leaving the PGE2-provoked and Smo-independent Hh activity in colorectal cancer cells unaffected. In this context, it is necessary to stratify the colorectal cancer patients by testing the PGE2 level of patients in prior to treatment. Combination of COX2 inhibitors with Smo inhibitors will be much more precise for colorectal cancer patients possessing aberrant PGE2 level and Hh activity. As the final effector of Hh pathway, Gli has emerged as a promising target for suppressing both canonical and non-canonical Hh activity. Numerous direct and indirect Gli inhibitors have been developed for treatment of Hh-driven cancers [43, 44]. However, it has been shown that Gli inhibitors function in a context-dependent manner [27]. This argument is consistent with our observations in this study. Our data reveals that Gli inhibitors JQ1, an epigenetic modulator which indirectly inhibits transcription of Gli by epigenetically targeting BRD4 [24], and GANT61 that interferes with the binding of Gli to the transcription site of target genes [23], suppresses the PGE2-provoked Hh activity and exhibited comparable inhibitory effect on the proliferation of colorectal cancer cells. However, As_2_O_3_, a Gli inhibitor that has been shown to inhibit Hh activity by decreasing Gli expression [25, 26], is unable to affect PGE2-provoked Hh activity, in despite of obvious inhibitory effect on the proliferation of colorectal cancer cells, possibly due to the its induction of apoptosis of cancer cells [31, 32]. Hence, our observations suggest that much more attention is needed, when choosing Gli inhibitors to modulate the Hh activity in colorectal cancer cells.

Ubiquitination, the covalent attachment of ubiquitin to substrate proteins, is a critical means to maintain the turnover of great majority of proteins in mammalian cells [45]. The interplay and crosstalk between phosphorylation and ubiquitination are a recurrent theme in eukaryotic biology. Phosphorylation can promote or inhibit ubiquitination, resulting in decreasing or increasing proteasomal degradation, respectively. In the context of inhibiting ubiquitination, it has been well recognized that phosphorylation of a substrate by a kinase may protect this substrate protein from ubiquitination and consequent degradation [46, 47]. Consistent with this paradigm, we found that Gli2 phosphorylation at Thr1546 caused by kinase JNK did endow Gli2 the ability of evading proteasomal-ubiquitin degradation. Hence, our study replenishes the paradigm that phosphorylation of a substrate by a kinase may protect this substrate protein from ubiquitination and consequent degradation, therefore, contributing to our better interpretation of the mechanisms underlying ubiquitination, which has emerged as a promising direction for developing molecular targeted anti-cancer drugs. In summary, this study demonstrate PGE2 non-canonically promotes Hh pathway activity by suppressing the ubiquitination of Gli2 through JNK, thereby promoting the progression of colorectal cancers. Hence, This study not only presents evidence for understanding the contribution of Hh to colorectal cancers, but also provides a novel molecular portrait underlying how PGE2-activated JNK fine-tunes the evasion of Gli2 from ubiquitin-proteasomal degradation. Therefore, it proposes a rationale for the future evaluation of chemopreventive and selective therapeutic strategies for colorectal cancers by targeting PGE2-JNK-Gli signaling route.

## Supplementary information

Supplemental figure 1

Supplemental figure 2

Supplemental figure 3

Supplemental figure 4

Supplemental figure 5

Supplemental figure legends

Supplemental tables
