# Supplementary figures and images for: PGE2-JNK signaling axis non-canonically promotes Gli activation by protecting Gli2 from ubiquitin-proteasomal degradation
Source: Cell Death Dis. 2021 Jul 15;12(7):707. doi: 10.1038/s41419-021-03995-z (PMC8282835; doi:10.1038/s41419-021-03995-z)

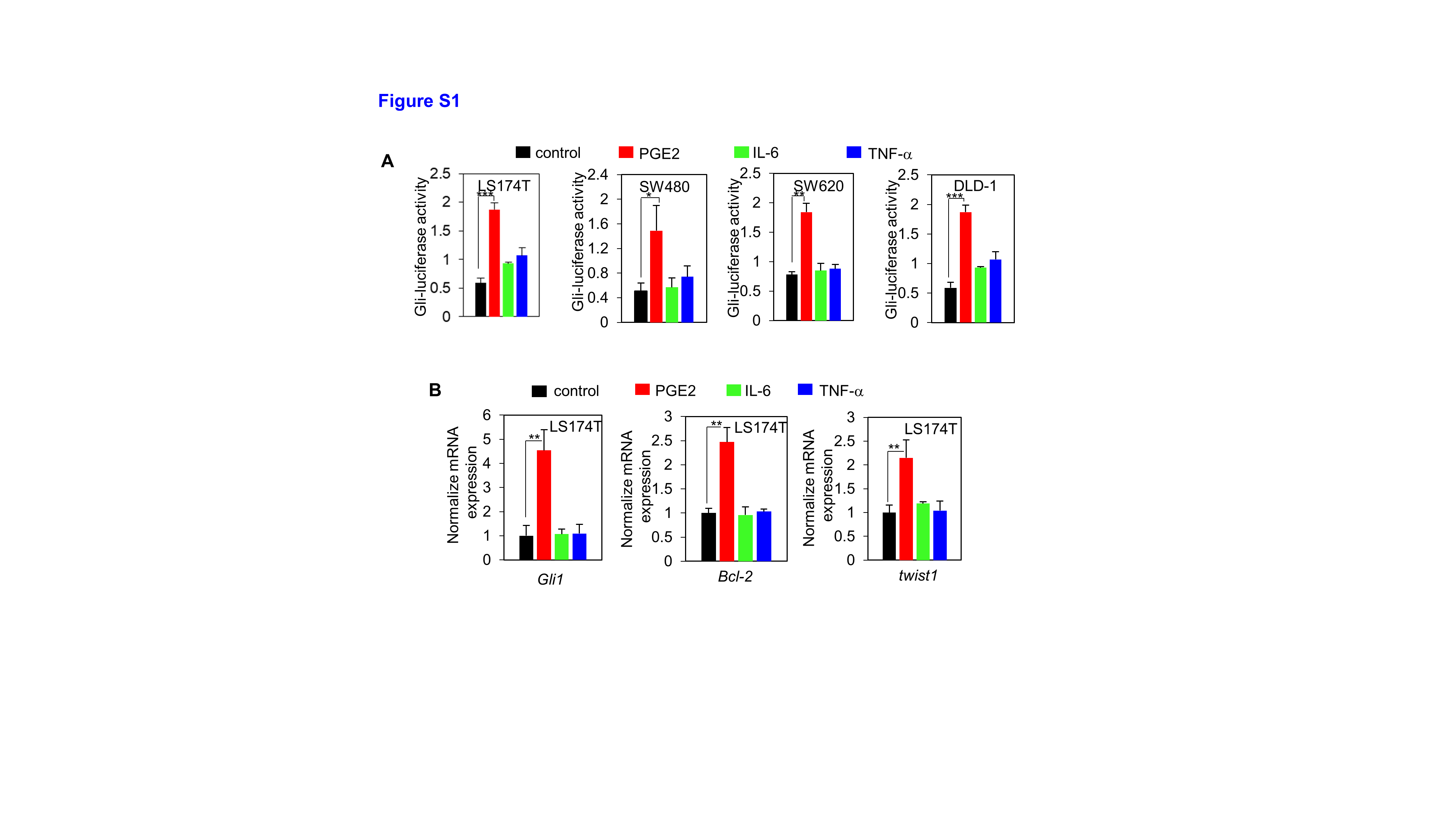

Supplement: Supplementary file 1 — Supplemental figure 1 [file 41419_2021_3995_MOESM1_ESM.tif]

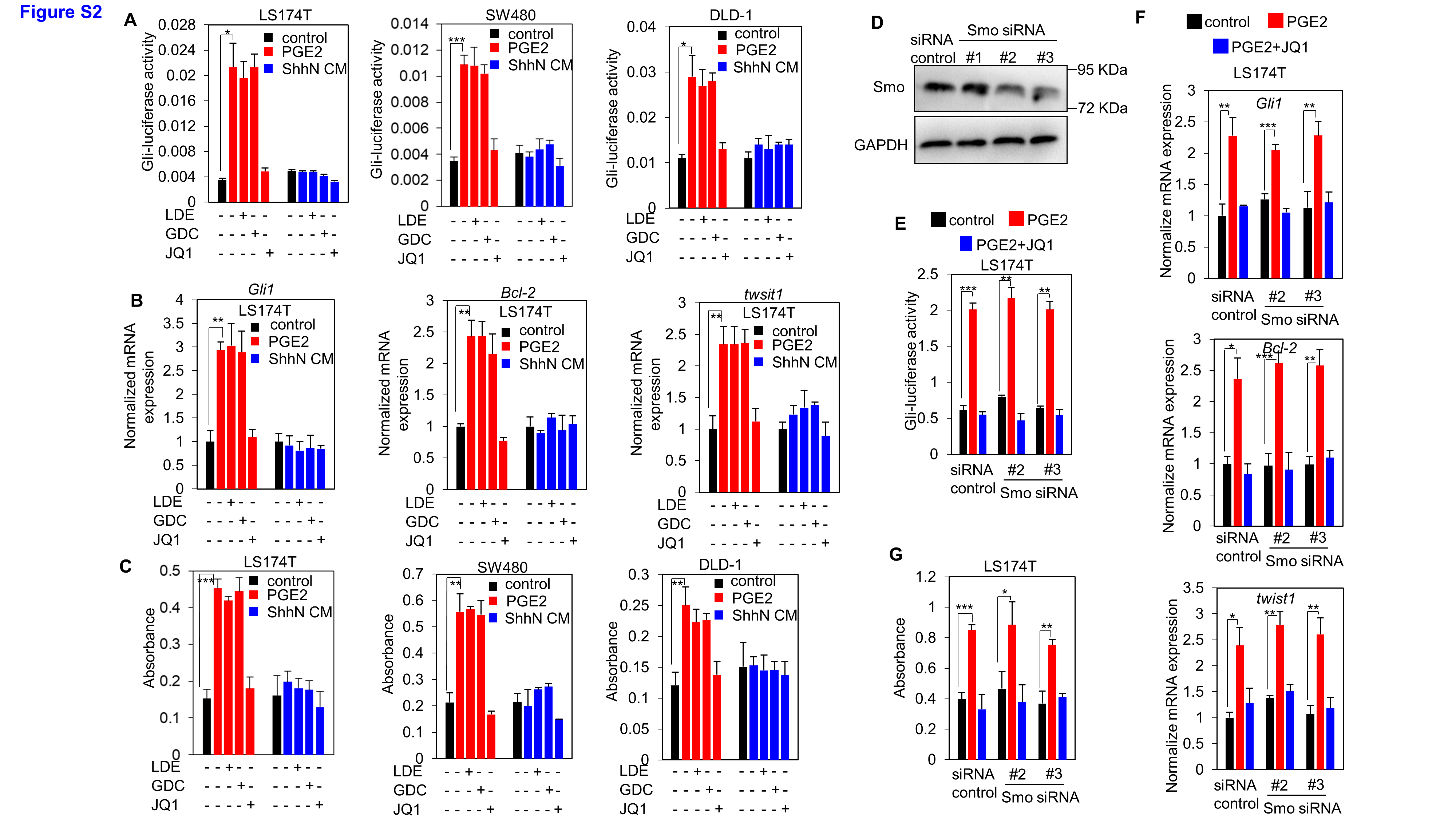

Supplement: Supplementary file 2 — Supplemental figure 2 [file 41419_2021_3995_MOESM2_ESM.tif]

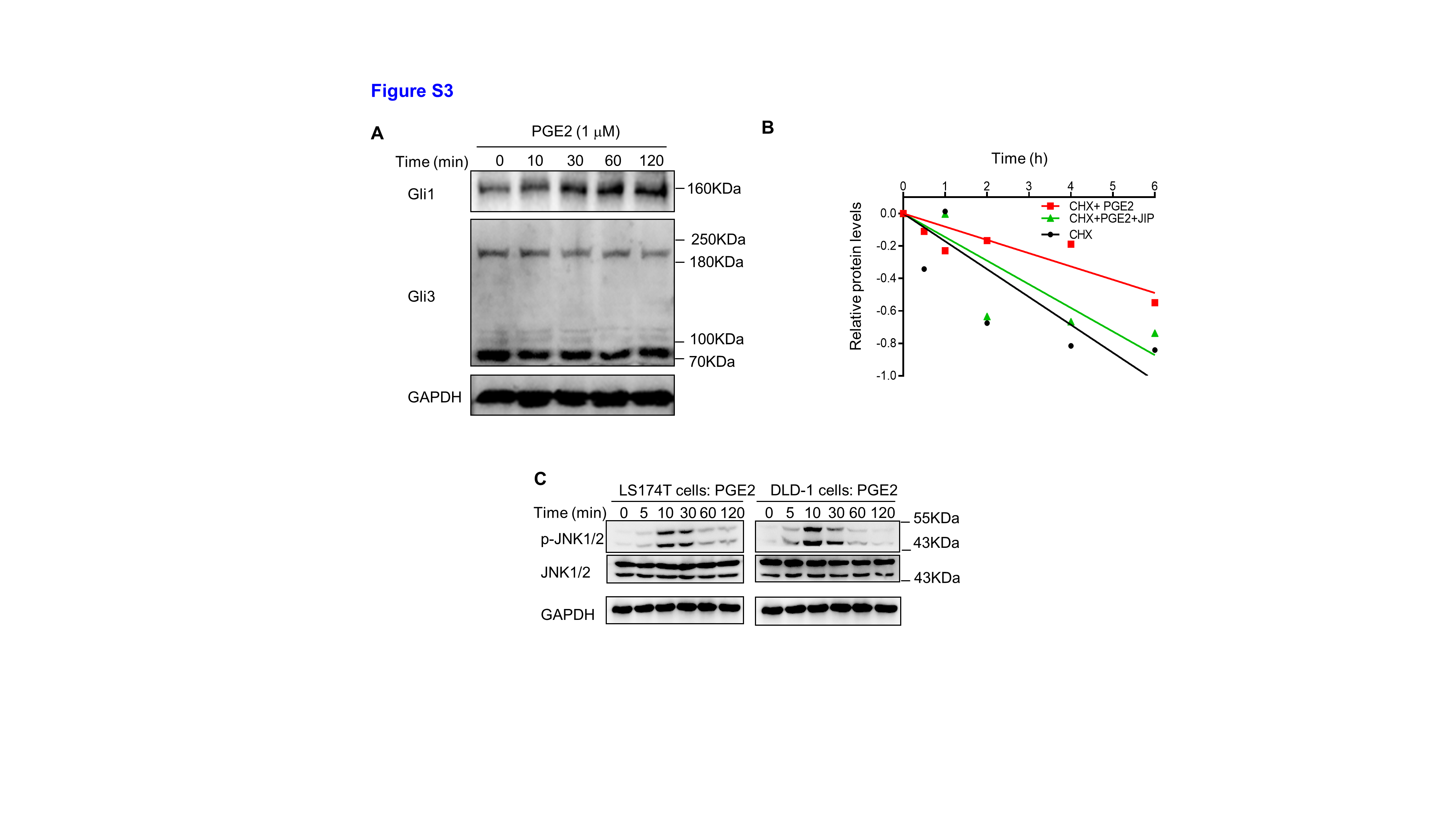

Supplement: Supplementary file 3 — Supplemental figure 3 [file 41419_2021_3995_MOESM3_ESM.tif]

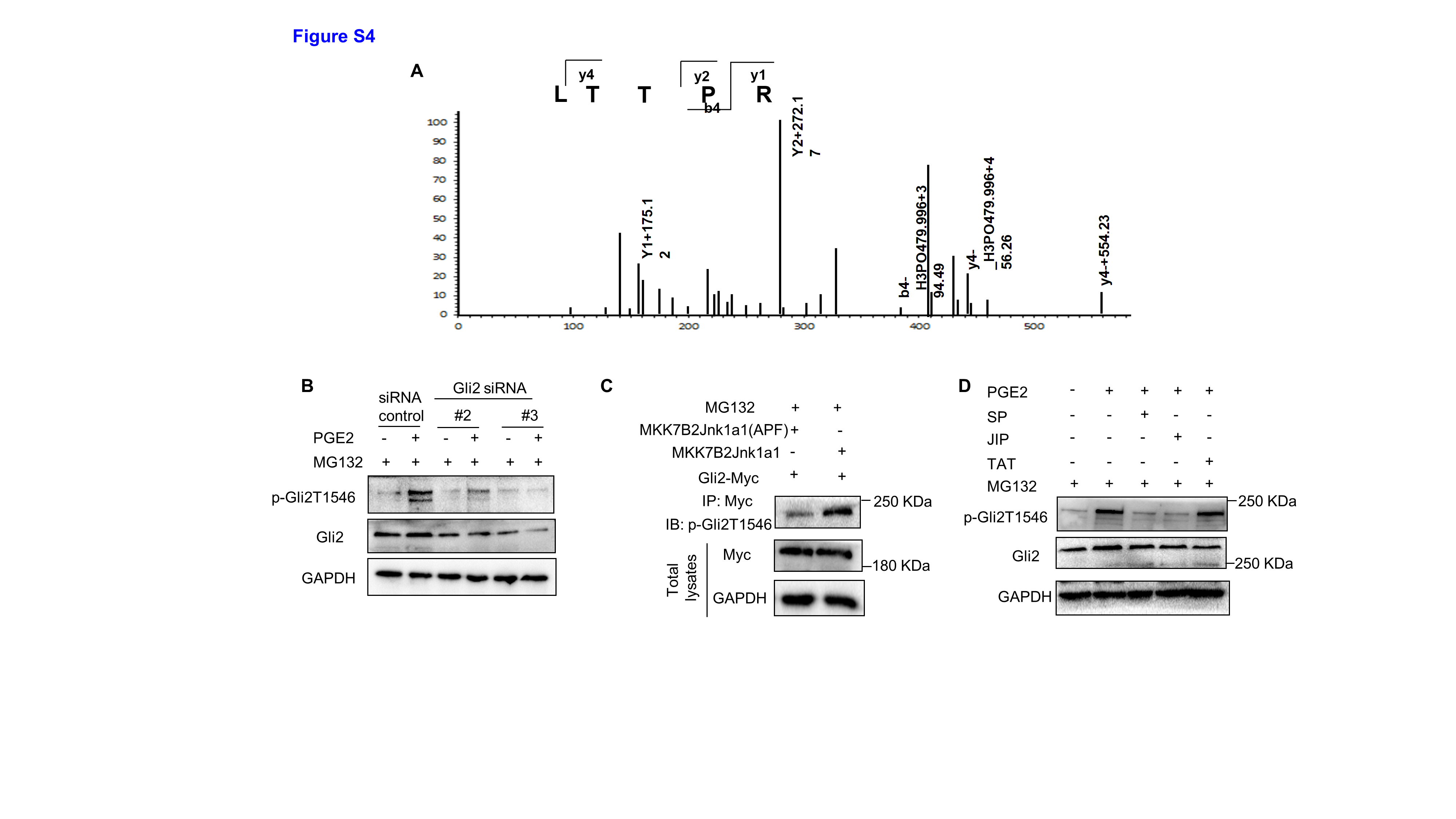

Supplement: Supplementary file 4 — Supplemental figure 4 [file 41419_2021_3995_MOESM4_ESM.tif]

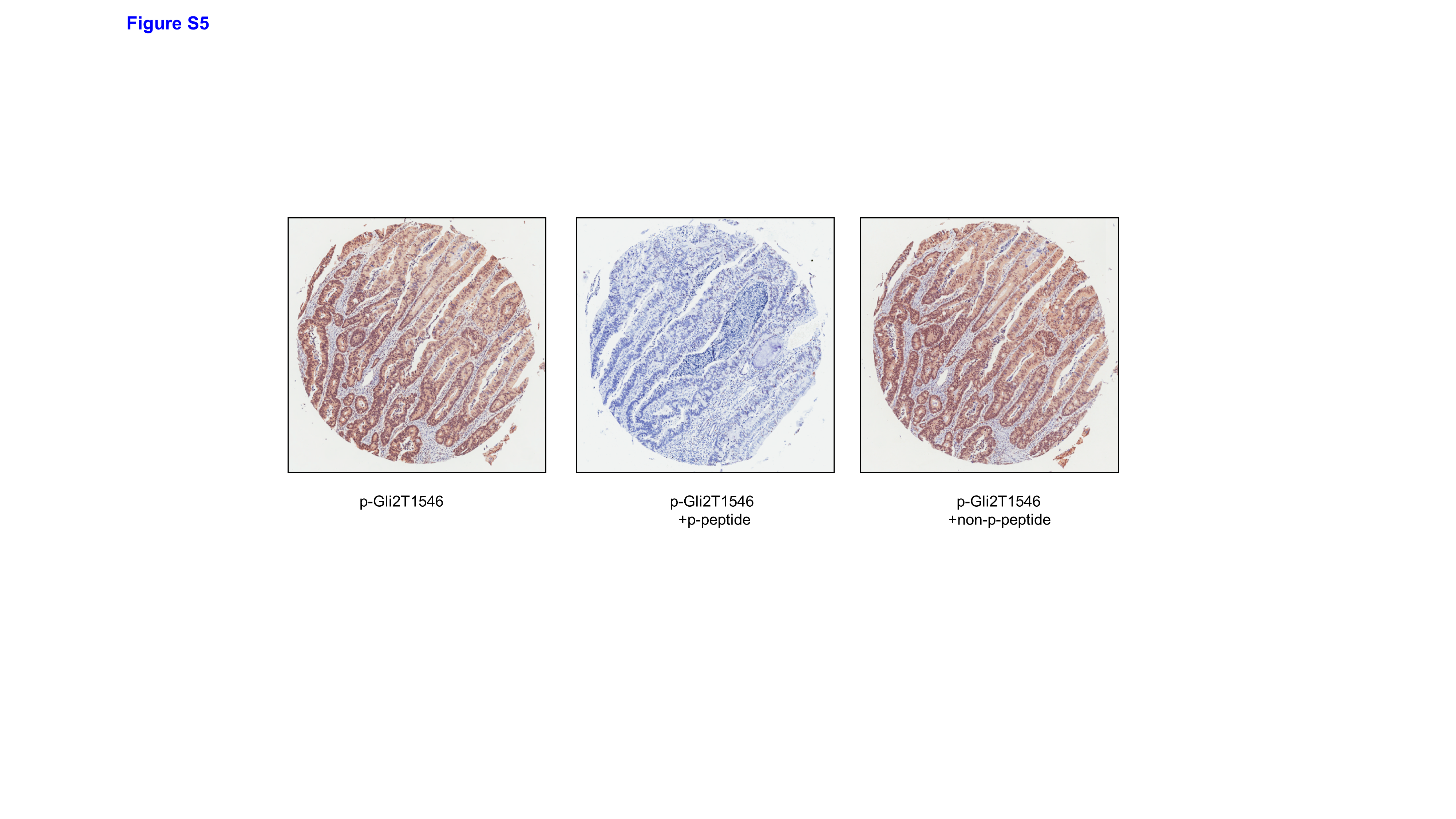

Supplement: Supplementary file 5 — Supplemental figure 5 [file 41419_2021_3995_MOESM5_ESM.tif]
