# Supplementary material for: PGE2-JNK signaling axis non-canonically promotes Gli activation by protecting Gli2 from ubiquitin-proteasomal degradation
Source: Cell Death Dis. 2021 Jul 15;12(7):707. doi: 10.1038/s41419-021-03995-z (PMC8282835; doi:10.1038/s41419-021-03995-z)
Supplement: Supplementary file 6 — Supplemental figure legends [file 41419_2021_3995_MOESM6_ESM.docx]

**Supplementary Figure Legends**

**Figure S1. TNF-α and IL-6 are unable to stimulate Hh activity in colorectal cancer cells. A** Luciferase assay for Gli transcriptional activity in colorectal cancer cells LS174T, SW480, SW620, and DLD-1 cells after exposure to PGE2, IL-1, and TNF-α as indicated for 6 h. Error bars represent SD (n = 3). **B** The mRNA levels of Gli target genes in LS174T cells after exposure to PGE2, IL-1, and TNF-α as indicated for 6 h were examined by real-time PCR and normalized to the mRNA level of *gusb*. Error bars represent SD (n = 3).

**Figure S2.** **Smo is dispensable for the activation of Hh activity by PGE2. A** Luciferase assay for Gli transcriptional activity in colorectal cancer cells treated by either PGE2 or ShhN CM in combination with or without Smo inhibitors LDE (100 nM), GDC (100 nM), and Gli inhibitor JQ1 (1 μM). Error bars represent SD (n = 3). **B** The mRNA levels of Gli target genes *Gli1*, *Bcl-2*, and *twist1* in LS174T cells were examined by real-time PCR and normalized to the mRNA level of *gusb*. LS174T cells were treated by either PGE2 or ShhN CM in combination with or without Smo inhibitors LDE (100 nM), GDC (100 nM), and Gli inhibitor JQ1 (1 μM) as indicated. Error bars represent SD (n = 3). **C** The proliferation of colorectal cancer cells subjected to either PGE2 or ShhN CM in combination with or without LDE (100 nM), GDC (100 nM), and JQ1 (1 μM) as indicated was examined by BrdU assays. Error bars represent SD (n = 3). **D** Expression of Smo protein in LS174T expressing Smo siRNAs and siRNA control was analyzed by western blotting. **E** Luciferase assay for Gli transcriptional activity in colorectal cancer cells LS174T expressing Smo siRNA #2 and #3 after exposure to PGE2 and Gli inhibitor JQ1 (1 μM) as indicated for 6 h. Error bars represent SD (n = 3). **F** The mRNA levels of Gli target genes in LS174T cells expressing Smo siRNA #2 and #3 after exposure to PGE2 and Gli inhibitor JQ1 as indicated for 6 h were examined by real-time PCR and normalized to the mRNA level of *gusb*. Error bars represent SD (n = 3). **G** The proliferation of colorectal cancer cells LS174T cells expressing Smo siRNA #2 and #3 after exposure to PGE2 and Gli inhibitor JQ1 as indicated for 6 h was examined with BrdU assays. Error bars represent SD (n = 3).

**Figure S3.** **PGE2 protects Gli2 from ubiquitin-proteasomal degradation by activating JNK. A** Expression of Gli1, and Gli3 protein in LS174T cells treated with PGE2 for different time intervals as indicated was analyzed by western blotting. **B** Densitometry results of Gli2 expression described in Figure 2C was plotted graphically. **c** Expression of p-JNK1/2, and JNK1/2 proteins in LS174T and DLD-1 cells treated with PGE2 for different time intervals as indicated were analyzed by western blotting.

**Figure S4. JNK activation by PGE2 protects Gli2 from degradation by phosphorylating Gli2 at Thr1546. A** Mass spectrometric analysis of recombinant JNK1-stimulated phosphorylation of recombinant Gli2-F3 fragment Thr1546. **B** Immunoblot analysis of Thr1546 phosphorylation of Gli2 in LS174T cells expressing siRNA control, and Gli2 siRNA after exposure to PGE2, MG132 as indicated for 1 h using specific phosphor-Gli2Thr1546 antibody. **C** Immunoprecipitation-western blot analysis of Thr1546 phosphorylation of Gli2 in LS174T cells expressing Myc-tagged Gli2, MKK7B2Jnk1a1(APF), MKK7B2Jnk1a1 after exposure to MG132 for 1 h using specific phosphor-Gli2Thr1546 antibody. **D** Immunoprecipitation-western blot analysis of Thr1546 phosphorylation of Gli2 in LS174T cells treated by PGE2, SP, JIP, TAT, and MG132 as indicated for 1 h using specific phosphor-Gli2Thr1546 antibody.

**Figure S5. Verifying the suitibility of the phospho-specific antibody for IHC analysis.** Immunohistochemistry analysis of p-Gli2T1546 antibody with phosphorylated Gli2 peptide or the matched nonphosphorylated Gli2 peptide.
