# Supplementary material for: PGE2-JNK signaling axis non-canonically promotes Gli activation by protecting Gli2 from ubiquitin-proteasomal degradation
Source: Cell Death Dis. 2021 Jul 15;12(7):707. doi: 10.1038/s41419-021-03995-z (PMC8282835; doi:10.1038/s41419-021-03995-z)
Supplement: Supplementary file 7 — Supplemental tables [file 41419_2021_3995_MOESM7_ESM.docx]

**Table S1. The sequence of siRNA.**

| siRNA Name | Sequence 5’-3’ |
| --- | --- |
| Gli2 siRNA#1 | UGAAUAAAGCAUCCAAGUAdTdT |
|  | UACUUGGAUGCUUUAUUCAdTdT |
| Gli2 siRNA#2 | GUGACACCAACCAGAACAAdTdT |
|  | UUGUUCUGGUUGGUGUCACdTdT |
| Gli2 siRNA#3 | GCACUGGCUUCUCUGACAAdTdT |
|  | UUGUCAGAGAAGCCAGUGCdTdT |
| Smo siRNA#1 | CCUGCGUCAUCAUCUUUGUdTdT |
|  | ACAAAGAUGAUGACGCAGGdTdT |
| Smo siRNA#2 | GUGGAGAAGAUCAACCUGUdTdT |
|  | ACAGGUUGAUCUUCUCCACdTdT |
| Smo siRNA#3 | CUGAUGGACACAGAACUCAdTdT |
|  | UGAGUUCUGUGUCCAUCAGdTdT |
| siRNA negative control | UUCUCCGAACGUGUCACGUdTdT |
|  | ACGUGACACGUUCGGAGAAdTdT |

**Table S2. Reagents used in this study.**

| Reagents | Source | Catalog # |
| --- | --- | --- |
| Prostaglandin E2 (PGE2) | Sigma-Aldrich | P5640 |
| As2O3 | ShuangLu Pharma | H20080665 |
| GANT-61 | Biovision | 1892-5 |
| JQ1 | Selleck Chemicals | S7110 |
| GDC-0449 | Selleck Chemicals | S1082 |
| LED-225 | MCE | HY-16582A |
| MG132 | Beyotime Biotechnology | S1748 |
| Chloroquine diphosphate salt | Sigma-Aldrich | C6628 |
| Cycloheximid (CHX) | MCE | HY-12320 |
| Rapamycin | Selleck Chemicals | S1039 |
| PD980059 | Selleck Chemicals | S1177 |
| SB203580 | Selleck Chemicals | S1076 |
| LY294002 | Selleck Chemicals | S1105 |
| Ro31-8220 | Selleck Chemicals | S7207 |
| SP600125 | Selleck Chemicals | S1460 |
| TAT-TI-JIP | Calbiochem | 420134 |
| TAT (JNK Inhibitor VI TI-JIP control) | Calbiochem | 420133 |
| JNK1a1/SAPK1c Protein with an N-terminal His-tag for kinase assay | Millipore | 14-327 |
| IL-6 | Peprotech | 200-06 |
| TNF-α | Peprotech | 300-01A |

**Table S3. Primers used in qPCR assay.**

| Gene | Primer |
| --- | --- |
| mGli1 forward | GCAGTGGGTAACATGAGTGTCT |
| mGli1 reverse | AGGCACTAGAGTTGAGGAATTGT |
| mBcl2 forward | GTCGCTACCGTCGTGACTTC |
| mBcl2 reverse | CAGACATGCACCTACCCAGC |
| mTwist1 forwaed | GGAGTCCGCAGTCTTACGAG |
| mTwist1 reverse | CCAGCTTGAGGGTCTGAATC |
| mGUSB forward | CTGCCACGGCGATGGA |
| mGUSB reverse | ACTGCATAATAATGGGCACTGTTG |
| hGli1 forward | GGGATGATCCCACATCCTCAGTC |
| hGli1 reverse | CTGGAGCAGCCCCCCCAGT |
| hBcl2 forward | GGTGAACTGGGGGAGGATTGT |
| hBcl2 reverse | CTTCAGAGACAGCCAGGAGAA |
| hTwist1 forward | CCAGGTCGTTTTTGAATGGT |
| hTwist1 reverse | ACGTGAGGAGGAGGGACTTT |
| hGUSB forward | TGGTTGGAGAGCTCATTTGGA |
| hGUSB reverse | GCACTCTCGTCGGTGACTGTT |

**Table S4. Antibodies used in this study.**

| Antibodies | Source | Catalog # |
| --- | --- | --- |
| Gli2 (R770) Antibody (WB) | CST | 2585 |
| Gli2 Antibody (IHC) | Novus Biologicals | NBP2-23602 |
| Smo (H-300) | Santa Cruz | SC-13943 |
| Myc-Tag (71D10) Rabbit mAb | CST | 2278 |
| HA-Tag (C29F4) Rabbit mAb | CST | 3724 |
| Gli1 (C68H3) Rabbit mAb | CST | 3538 |
| Gli3 (H-280) | Santa Cruz | SC-20688 |
| Phospho-SAPK/JNK (Thr183/Tyr185) (81E11) Rabbit mAb (WB) | CST | 4668 |
| Human/Mouse/Rat Phospho-JNK (T183/Y185) Antibody (IHC) | R&D Systems | AF1205 |
| JNK2 (56G8) Rabbit mAb | CST | 9258 |
| DYKDDDDK Tag Antibody (Binds to same epitope as Sigma's Anti-FLAG® M2 Antibody) | CST | 2368 |
| Purified Mouse Anti-Phosphoserine/threonine Clone 22A/pSer/Thr (RUO) | BD | 612548 |
| p-Gli2(T1546) (IHC)* | Abmart | NA |
| Anti-Ki67 (SP6) | Abcam | ab16667 |
| GAPDH(G-9) | Santa Cruz | SC-365062 |
| ProteinA/G plus-Agarose | Santa Cruz | SC-2003 |
| Peroxidase-labeled  goat anti-rabbit | Dingguo Changsheng Biotchnology | IH0011 |
| Peroxidase-labeled  goat anti-mouse | Dingguo Changsheng Biotchnology | IH-0031 |

*****It was produced using the synthetic phosphorylated peptide (CSSRLTT(p)PRN)
